# Morphological characterization of wild *Prunus scoparia* Spach accessions in 11 provinces of Iran

**DOI:** 10.1038/s41598-023-43146-2

**Published:** 2023-09-22

**Authors:** Ali Khadivi, Farhad Mirheidari, Younes Moradi

**Affiliations:** Department of Horticultural Sciences, Faculty of Agriculture and Natural Resources, Arak University, Arak, 38156-8-8349 Iran

**Keywords:** Ecology, Evolution, Genetics, Physiology, Plant sciences, Structural biology

## Abstract

*Prunus scoparia* (Spach) C. K. Schneid is among the most prevalent species which has the potential of being used as a dwarf rootstock for the cultivated almond. In the present study, the phenotypic variation of 521 wild accessions of this species naturally grown in 29 areas of 11 provinces in Iran was assessed. The accessions investigated showed significant differences based on the measured traits. The majority of the characters measured (90 out of 100) exhibited a coefficient of variation of higher than 20.00%, indicating considerable variation among the accessions. The range of nut-related characters was as follows: nut length: 9.72–22.87 mm, nut width: 5.81–15.54 mm, nut thickness: 5.67–12 mm, and nut weight: 0.18–0.99 mm. The range of kernel-related characters was as follows: kernel length: 6.83–19.23 mm, kernel width: 4.28–10.32 mm, kernel thickness: 2.16–7.52 mm, and kernel weight: 0.03–0.37 g. Kernel weight exhibited positive and significant correlations with nut length (*r* = 0.57), nut width (*r* = 0.54), nut thickness (*r* = 0.42), nut weight (*r* = 0.69), kernel length (*r* = 0.75), kernel width (*r* = 0.78), and kernel thickness (*r* = 0.58). Cluster analysis based on Ward’s method showed two different major clusters among all the accessions. Based on the bi-plot created using principal component analysis of population analysis, the studied 29 natural habitats formed four groups. The studied accessions showed considerable variation in terms of the measured traits within and among populations. This variation is due to cross-pollination, cross-incompatibility, natural hybridization, propagation by seeds, gene flow, and exchange of plant material between the study areas. By using crosses between accessions of different regions, it is possible to increase the amount of variability in different traits of wild almonds.

## Introduction

Iran is located in arid and semi-arid areas, and more than 60% of this country includes these areas. Wild species are a valuable genetic resource in terms of desired traits in breeding programs, which include traits related to trees and fruits. Accordingly, using plants resistant to such weather conditions should be prioritized^1^. Also, the valuable features of resistance to biotic and abiotic stresses are evident in these species, which can be used in breeding programs to improve domesticated plants^2^. The diversity of wild species of fruit trees in Iran is considerable, and there are reserves rich in more almond species than 20 species, whose distribution has been reported here, some of which are endemic^2,3^.

Some of the wild species of almonds can have high survival in water shortage due to having some characteristics, such as defoliation during the hot season and high ability in absorption and storage of water, and the useful features of drought resistance in them can be evident in breeding programs^4^. Also, these species grow in shallow and rocky soils, and sometimes they grow in rocks^5^. Wild species of almonds have been used in Iran since 300 years ago as a rootstock for almonds or related species^6^. In different regions of Iran, including Hormozgan, Bushehr, Kerman, and Fars provinces, there are many orchards of almonds that are grafted onto wild-related species^2^. Wild almond species can be used in economics and ecology. Their kernels and oil are used by local people. In addition, wild almond species play a role of physical soil protection and have a high ability to prevent soil erosion^3,7^.

The resistance of wild almond species to hot and dry weather conditions as well as salinity and cold stress is high^8^. Other important characteristics of this valuable gene pool are late late-blooming, self-fertilization, and dwarfing^9^. Therefore, this important genetic resource can be used in future breeding programs, landscape, and reforestation. They can also be used for air purification in polluted areas^9–11^.

**Figure 1:**
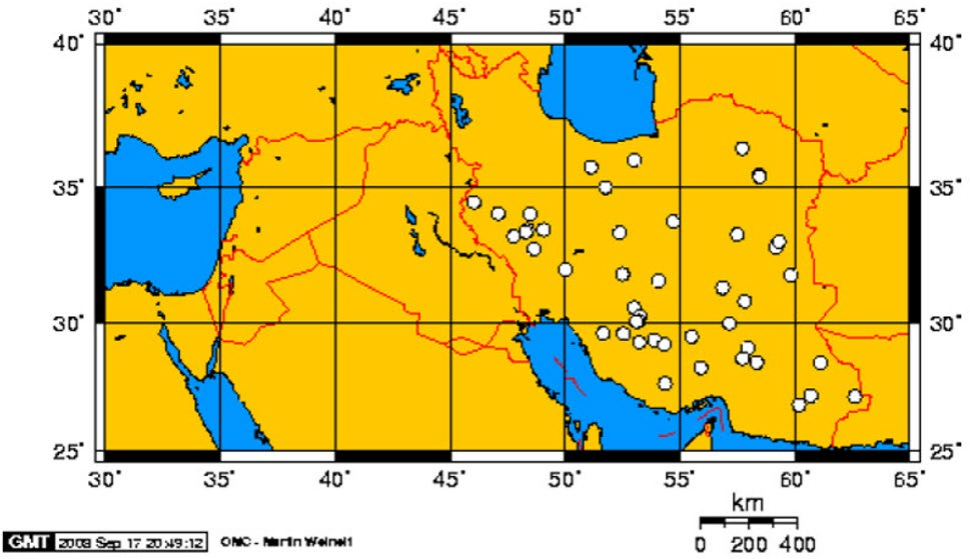
Geographic distribution of *P. scoparia* in Iran^12^.

One of the wild almond species that is widely distributed throughout Iran is *Prunus scoparia* (Spach) C. K. Schneid (Fig. 1)^12^. This wild species has multi-purpose importance, the most important of which is its use as a rootstock for domesticated almonds^7^. It can also be used to stabilize and prevent soil erosion in arid and semi-arid regions^13^. High resistance to drought stress and infertile soils, dense and green canopy, beautiful flowers, long-lasting green branches, and long flowering period make this plant a suitable choice for the landscape in arid and semi-arid areas, especially in mountains around cities^3,7^.

Successful plant breeding programs are highly dependent on their genetic diversity. Investigating and determining genetic diversity is very important. Wild species are valuable genetic resources that are abundant and have a high potential for crop improvement in breeding programs. Therefore, identifying their promising accessions is needed^9^. Analysis of morphological traits is one of the first steps and the most common methods for germplasm description and identification. In the present study, the phenotypic variation of 521 wild accessions of *P. scoparia* collected from 29 regions of 11 provinces in Iran was evaluated. The findings of the present study can contribute to effective breeding programs.

## Materials and methods

### Plant material

The phenotypic variation of 521 wild accessions of *P. scoparia* collected from 29 regions of 11 provinces in Iran was evaluated for two consecutive years (2021 and 2022). Table 1 contains the geographical characteristics of the studied areas. The identification of the specimens was performed by Prof. Dr. Ali Khadivi. A herbarium voucher specimen with sediment number PS-2443 was donated to a public available herbarium of the Faculty of Agriculture and Natural Resources of Arak University, Iran. Permits required to collect the studied plant samples were obtained from the Ministry of Agriculture and Natural Resources of Iran. For correct sampling, a proper distance of at least 200 m between the accessions of each area was regarded so that the clone samples were not collected.

**Table 1:** Geographical description for collection sites of *P. scoparia* accessions studied in Iran.

No	Province	Area	Abbreviation	Longitude (E)	Latitude (N)	Altitude (m)	Sample size
1	Isfahan	Tangestan	T	52° 56′ 22″	32° 45′ 38″	1978	17
2	Isfahan	Kapise	K	51° 23′ 27″	32° 27′ 31″	1785	20
3	Isfahan	Rokh	R	51° 04′ 52″	32° 20′ 35″	2149	15
4	Isfahan	Moorchekhort	M	51° 27′ 26″	33° 06′ 31″	1689	20
5	Isfahan	Barzok	B	51° 13′ 18″	33° 50′ 23″	1715	20
6	Isfahan	Alvar	A	50° 54′ 15″	32° 50′ 51″	2113	15
7	Tehran	Saidabad	S	51° 42′ 09″	35° 43′ 14″	1450	21
8	Khuzistan	Babamir	Ba	50° 22′ 39″	31° 13′ 15″	920	23
9	Khuzistan	Sarrak	Sa	50° 25′ 43″	31° 34′ 12″	935	21
10	Khuzistan	Sargach	Sg	49° 44′ 36″	32° 20′ 44″	780	17
11	Fars	Kelestan	Ke	52° 13′ 30″	29° 56′ 02″	2271	20
12	Fars	Maharlo	Ma	52° 46′ 05″	29° 26′ 21″	1445	20
13	Fars	Hajiabad	H	54° 08′ 27″	29° 11′ 22″	1627	20
14	Fars	Bahramgoor	Bg	54° 33′ 07″	29° 13′ 00″	1854	20
15	Qom	Zavarian	Z	50° 24′ 47″	34° 26′ 40″	1485	15
16	Qom	Esfid	E	50° 26′ 07″	34° 33′ 35″	1434	15
17	Qom	Hesarsorkh	He	50° 29′ 34″	34° 22′ 10″	1345	15
18	Kurdistan	Shilan	Sh	46° 56′ 01″	35° 04′ 59″	1340	13
19	Kerman	Chahbot	Ch	55° 34′ 08″	29° 17′ 17″	1699	20
20	Kerman	Sharbabak	Sb	55° 23′ 10″	29° 51′ 52″	1863	20
21	Lorestan	Shoorab	So	48° 12′ 31″	33° 26′ 05″	1163	9
22	Markazi	Zavieh	Za	50° 56′ 56″	35° 38′ 31″	1320	30
23	Markazi	Kheirabad	Kr	49° 57′ 50″	34° 07′ 56″	1705	15
24	Hormozgan	Hajiabad	Hj	55° 54′ 32″	28° 18′ 26″	955	15
25	Baluchestan	Bazman	Bz	60° 29′ 44″	28° 40′ 11″	945	15
26	Baluchestan	Khash	Ks	61° 21′ 58″	28° 22′ 27″	1415	15
27	Yazd	Zarju	Zj	53° 36′ 17″	32° 20′ 57″	1499	20
28	Yazd	Taft	Tf	54° 13′ 15″	31° 43′ 03″	1692	20
29	Yazd	Kalmand	Km	54° 48′ 32″	31° 18′ 25″	1588	15

### The characters evaluated

In total, 100 morphological traits related to flowers, branches, leaves, and fruits were recorded using 50 replications for each organ. Traits related to the size and weight of different organs were measured through digital calipers and electronic scales, respectively. To estimate the qualitative attributes in the form of code and rank, the almond descriptor (IPGRI) was used^14^.

### Statistical analysis

The average data were used for analyses. To determine the significance between accessions, analysis of variance was done using SAS software^15^. The SPSS software^16^ was used to determine the correlation between the traits as well as principal component analysis (PCA). Cluster analysis based on Ward's method and Euclidean distance and creating a scatter plot based on PC1 and PC2 were done using PAST software^17^.

### Statement specifying permissions

For this study, we acquired permission to collect *P. scoparia* specimens issued by the Agricultural and Natural Resources Ministry of Iran.

### Statement on experimental research and field studies on plants

All methods performed on plants (either cultivated or wild), including the collection of plant material comply with relevant institutional, national, and international guidelines and domestic legislation of Iran.

## Results and discussion

The accessions investigated showed significant differences based on the traits (ANOVA, *P* < 0.01). The majority of the characters (90 out of 100) exhibited a coefficient of variation (CV) of higher than 20.00%, indicating considerable variation among the accessions. The lowest CVs belonged to nut thickness (11.12%), sepal shape (14.43%), nut width (14.43%), current branch leaf apex shape (14.76%), and kernel width (15.57%), while suture opening of the shell showed the highest CV (685%), followed by the marking of the outer shell (171.28%), fruit stigma retention (161.07%), kernel shriveling (136.80%), shell surface pubescent (136.38%), shell ornamentation (133.33%), shell ornamentation present (130.27%), shell back line shape (117.34%), peduncle color (110.96%), and shell short furrows starting from the base (106.38%) (Table 2). Variation in the species and cross-pollination that lead to heterozygosity and increased genetic diversity in almonds during development and evolution can increase the CV value of different traits among accessions^18^.

**Table 2:** Statistical descriptive parameters for morphological traits used to study *P. scoparia* accessions.

No	Trait	Abbreviation	Unit	Min	Max	Mean	SD	CV (%)
1	Flowering date	FlD	Code	1	9	4.02	2.41	59.83
2	Flower density	FlDe	Code	1	5	3.79	1.38	36.33
3	Peduncle color	PedCo	Code	1	9	2.29	2.54	110.96
4	Peduncle length	PedLe	mm	0.77	3.95	2.28	0.53	23.22
5	Peduncle width	PedWi	mm	0.95	3.36	1.62	0.39	24.03
6	Petal color	PetCo	Code	1	7	1.81	1.37	75.58
7	Petal shape	PetSh	Code	1	7	4.34	1.85	42.56
8	Petal apex shape	PetApSh	Code	1	7	3.50	0.96	27.34
9	Petal length	PetLe	mm	4.30	16.37	9.66	2.04	21.11
10	Petal width	PetWi	mm	2.56	15.01	7.81	2.44	31.31
11	Hypanthium color	HyCo	Code	1	9	4.61	2.03	44.01
12	Hypanthium length	HyLe	mm	1.09	7.65	3.59	0.83	23.08
13	Hypanthium diameter	HyDi	mm	1.12	6.81	4.00	1.04	26.00
14	Sepal shape	SepSh	Code	1	3	2.91	0.42	14.43
15	Sepal apex shape	SepApSh	Code	1	5	4.02	1.08	26.74
16	Sepal external color	SepExCo	Code	3	11	7.50	2.04	27.21
17	Sepal internal color	SepInCo	Code	1	9	5.22	2.53	48.51
18	Sepal length	SepLe	mm	1.50	6.66	3.81	0.90	23.55
19	Sepal width	SepWi	mm	0.60	5.65	2.73	0.72	26.41
20	Number of stamens	StNo	Number	3	36	26.65	5.59	20.98
21	Stamen color	StCo	Code	1	7	2.32	1.35	57.97
22	Carpel number	CaNo	Number	1	3	1.12	0.47	41.61
23	Stigma length	StLe	mm	1.58	10.53	5.46	1.45	26.50
24	Carpel shape	CaSh	Code	1	5	1.18	0.72	61.27
25	Tree form	TrFo	Code	1	5	1.63	1.33	81.60
26	Tree growth habit	TrGrHa	Code	1	9	6.19	1.90	30.69
27	Tree growth vigor	TrGrVi	Code	1	5	3.72	1.42	38.17
28	Tree height	TrHe	Code	1	7	3.20	1.73	53.97
29	Trunk color intensity	TruCo	Code	1	15	6.73	4.16	61.83
30	Trunk type	TruTy	Code	1	7	5.50	1.94	35.29
31	Trunk diameter	TrDi	Code	1	5	2.43	1.56	64.36
32	Canopy density	CaDe	Code	1	5	3.21	1.51	47.01
33	Branching	Br	Code	1	5	3.52	1.30	36.79
34	Branch density	BrDe	Code	1	7	3.50	1.47	41.86
35	Branch flexibility	BrFl	Code	1	5	4.43	1.07	24.11
36	Leaf density	LDe	Code	0	5	2.16	1.51	69.72
37	Annual branch leaf length	AnBrLLe	mm	8.11	54.35	22.54	6.95	30.85
38	Annual branch leaf width	AnBrLWi	mm	1.23	10.15	3.54	1.66	47.01
39	Annual branch petiole length	AnBrPetLe	mm	0.60	9.55	4.34	1.82	41.91
40	Annual branch petiole width	AnBrPetWi	mm	0.25	1.10	0.59	0.17	28.81
41	Annual branch leaf shape	AnBrLSh	Code	1	7	1.95	1.35	69.08
42	Annual branch leaf edge form	AnBrEdFo	Code	1	5	1.66	1.08	64.88
43	Annual branch leaf serration shape	AnSeShAnL	Code	1	7	4.82	1.77	36.66
44	Annual branch leaf serration depth	AnSeDepAnBr	Code	0	5	1.64	1.15	70.12
45	Annual branch leaf upper surface color	AnBrUCo	Code	1	5	3.05	1.01	32.98
46	Annual branch leaf lower surface color	AnBrLoCo	Code	1	5	1.67	1.01	60.30
47	Annual branch leaf vein color	AnBrVCo	Code	1	7	3.10	2.00	64.52
48	Annual branch leaf apex shape	AnBrLAp	Code	1	3	2.85	0.52	18.28
49	Transparency of current branch bark	TrSkSp	Code	1	3	1.43	0.82	57.48
50	Current branch leaf length	CuLLe	mm	8.00	40.25	20.14	5.21	25.88
51	Current branch leaf width	CuLWi	mm	0.74	10.15	2.94	1.21	41.26
52	Current branch petiole length	CuPetLe	mm	0.46	10.31	4.10	1.75	42.56
53	Current branch petiole width	CuPetWi	mm	0.30	0.91	0.56	0.15	26.07
54	Current branch leaf shape	CuLSh	Code	1	7	1.56	1.02	65.26
55	Current branch leaf edge form	CuLEdFo	Code	1	5	1.58	1.01	63.61
56	Current leaf serration shape	CuSeShL	Code	1	7	4.72	1.85	39.19
57	Current leaf serration depth	CuSeDep	Code	0	5	1.42	1.05	73.94
58	Current leaf upper surface color	CuUCo	Code	1	5	2.74	1.13	41.24
59	Current leaf lower surface color	CuLoCo	Code	1	5	1.72	0.98	57.21
60	Current branch leaf apex shape	CuBrLap	Code	1	3	2.90	0.43	14.76
61	Fruit yield	Yi	Code	1	5	3.02	1.52	50.20
62	Ripening date	RiD	Code	1	13	6.30	4.19	66.48
63	Fruit pubescence	FrPu	Code	0	5	1.98	1.51	76.41
64	Fruit stalk length	FrStaLe	mm	1.85	5.53	3.30	0.64	19.33
65	Fruit stalk diameter	FrStaDi	mm	0.88	3.48	2.00	0.51	25.55
66	Fruit skin color	FrSkCo	Code	1	27	9.57	6.77	70.70
67	Exocarp thickness	ExTh	Code	1	5	2.29	1.20	52.25
68	Exocarp splitting	ExSp	Code	0	1	0.93	0.26	27.63
69	Fruit skin retention	FrSkRet	Code	0	1	0.51	0.50	98.04
70	Fruit stigma retention	FrStRet	Code	0	1	0.28	0.45	161.07
71	Nut apex shape	NuApSh	Code	1	5	3.38	1.68	49.56
72	Nut base shape	NuBaSh	Code	1	5	2.30	1.82	78.96
73	Nut symmetry	NuSy	Code	0	1	0.83	0.38	45.78
74	Nut shape	NuSh	Code	1	15	9.51	3.70	38.85
75	Position of maximum transverse diameter	PMTDi	Code	1	5	3.33	1.13	33.90
76	Shell surface pubescence	ShePu	Code	0	5	0.94	1.28	136.38
77	Nut length	NuLe	mm	9.72	22.87	13.92	2.48	17.85
78	Nut width	NuWi	mm	5.81	15.54	9.84	1.42	14.43
79	Nut thickness	NuTh	mm	5.67	12.00	7.70	0.86	11.12
80	Nut weight	NuWe	g	0.18	0.99	0.48	0.17	36.25
81	Shell hardness	SheHar	Code	1	5	3.68	1.31	35.60
82	Shell color intensity	SheCo	Code	1	9	5.87	2.35	39.98
83	Shell thickness	SheTh	mm	0.40	1.76	1.06	0.24	22.41
84	Suture opening of the shell	SheOp	Code	0	1	0.02	0.14	685.00
85	Shell ornamentation present	SheOr	Code	0	1	0.37	0.48	130.27
86	Marking of outer shel	MaOu	Code	0	5	0.78	1.34	171.28
87	Shell ornamentation	SheOr	Code	0	1	0.36	0.48	133.33
88	Shell abdominal line shape	SheAbLiSh	Code	1	5	1.79	1.22	68.38
89	Shell back line shape	SheBaLi	Code	1	9	1.88	2.21	117.34
90	Shell abdominal line color	SheAbLi	Code	1	13	6.96	3.37	48.35
91	Shell back line color	SheBaCo	Code	1	13	7.02	3.09	43.96
92	Shell short furrows starting from base	SheSho	Code	0	1	0.47	0.50	106.38
93	Kernel length	KeLe	mm	6.83	19.23	10.99	2.02	18.42
94	Kernel width	KeWi	mm	4.28	10.32	6.66	1.04	15.57
95	Kernel thickness	KeTh	mm	2.16	7.52	4.57	0.83	18.07
96	Kernel weight	KeWe	g	0.03	0.37	0.16	0.07	43.75
97	Kernel shape	KeSh	Code	1	15	9.21	2.49	27.02
98	Kernel color intensity	KeCo	Code	1	7	3.51	1.10	31.37
99	Kernel shriveling	KeShr	Code	0	5	1.03	1.41	136.80
100	Kernel taste	KeTa	Code	3	9	3.89	1.22	31.29

Peduncle length ranged from 0.77 to 3.95 mm, while peduncle width varied from 0.95 to 3.36 mm. Petal length varied from 4.30 to 16.37 mm, and petal width ranged from 2.56 to 15.01 mm. Sepal length ranged from 1.50 to 6.66 mm, while sepal width varied between 0.60 and 5.65 mm.

Tree height was moderate (1–2 m) and then low (< 1 m) in the majority of accessions (263 and 125 accessions) (Table 3). In breeding programs, low tree height is considered a useful trait for introducing dwarfing rootstocks^7,19^.

**Table 3:** Frequency distribution for the measured qualitative morphological characters in the studied *P. scoparia* accessions.

Character	Frequency (no. of accessions)								
	0	1	3	5	7	9	11	13	15
Flowering date	–	Early March (146)	Mid-March (105)	Late March (151)	Early April (96)	Mid-April (23)	–	–	–
Flower density	–	Low (61)	Moderate (193)	High (267)	–	–	–	–	–
Peduncle color	–	Light green (400)	Green (10)	Green-crimson (51)	Light crimson (17)	Crimson (43)		–	–
Petal color	–	White (369)	White-pink (95)	Light pink (56)	Light pink (56)	Pink (1)	–	–	–
Petal shape	–	Oblate (34)	Round (235)	Obovat (122)	Oblong (130)	–	–	–	–
Petal apex shape	–	Falt (9)	Round (376)	Semi-round (134)	Sharp (2)	–	–	–	–
Hypanthium color	–	Light green (17)	Green-crimson (258)	Light crimson (74)	Crimson (153)	Dark crimson (19)	–	–	–
Sepal shape	–	Equilateral (24)	Equivalent of the legs (497)	–	–	–	–	–	–
Sepal apex shape	–	Round (10)	Semi-round (234)	Acute (277)	–	–	–	–	–
Sepal external color	–	–	Green (9)	Green-crimson (164)	Light crimson (67)	Crimson (250)	Dark crimson (31)	–	–
Sepal internal color	–	Light green (113)	Green (8)	Green-crimson (152)	Light crimson (204)	Crimson (44)	–	–	–
Stamen color	–	White (218)	Green-crimson (282)	Light crimson (2)	Crimson (19)	–	–	–	–
Carpel shape	–	Jar (488)	Filamentary (20)	Triangular (13)	–	–	–	–	–
Tree form	–	Shrub (417)	Small tree (45)	Tree (59)	–	–	–	–	–
Tree growth habit	–	Erect (10)	Semi-erect (46)	Open (185)	Spreading (184)	Weeping (96)	–	–	–
Tree growth vigor	–	Low (71)	Moderate (191)	High (259)	–	–	–	–	–
Tree height	–	Low (125)	Moderate (263)	High (88)	Very high (45)	–	–	–	–
Trunk color intensity	–	Light brown (43)	Brown (96)	Dark brown (177)	Black-brown (47)	Gray (34)	Dark gray (8)	Brown-gray (87)	Gray-black (29)
Trunk type	–	Single-trunk (39)	Multi-trunk/low (79)	Multi-trunk/moderate (116)	Multi-trunk/high (287)	–	–	–	–
Canopy density	–	Low (123)	Moderate (219)	High (179)	–	–	–	–	–
Branching	–	Low (59)	Moderate (268)	High (194)	–	–	–	–	–
Branch density	–	Low (90)	Moderate (213)	High (217)	Very high (1)	–	–	–	–
Branch flexibility	–	Low (21)	Moderate (107)	High (393)	–	–	–	–	–
Leaf density	Absent (48)	Low (212)	Moderate (195)	High (66)	–	–	–	–	–
Annual branch leaf shape	–	Narrow-lanceolate (326)	lanceolate (144)	Broad-lanceolate (50)	oblong (1)	–	–	–	–
Annual branch leaf edge form	–	Smooth (368)	Studs (135)	Curly (18)	–	–	–	–	–
Annual branch leaf serration shape	–	Entire (39)	Undulate (111)	Crenate (228)	Serrate (143)	–	–	–	–
Annual branch leaf serration depth	Absent (38)	Low (312)	Moderate (156)	High (15)	–	–	–	–	–
Annual branch leaf upper surface color	–	Light green (59)	Green (389)	Dark green (73)	–	–	–	–	–
Annual branch leaf lower surface color	–	Light green (355)	Green (158)	Dark green (8)	–	–	–	–	–
Annual branch leaf vein color	–	Light green (240)	Cream-green (24)	Green (249)	Dark green (8)	–	–	–	–
Annual branch leaf apex shape	–	Ronud (38)	Acute (483)	–	–	–	–	–	–
Transparency of current branch bark	–	Matt (409)	Transparent (112)	–	–	–	–	–	–
Current branch leaf shape	–	Narrow-lanceolate (390)	lanceolate (118)	Broad-lanceolate (12)	oblong (1)	–	–	–	–
Current branch leaf edge form	–	Smooth (382)	Studs (127)	Curly (12)	–	–	–	–	–
Current leaf serration shape		Entire (54)	Undulate (101)	Crenate (230)	Serrate (136)	–	–	–	–
Current leaf serration depth	Absent (54)	Low (338)	Moderate (121)	High (8)	–	–	–	–	–
Current leaf upper surface color	–	Light green (122)	Green (346)	Dark green (53)	–	–	–	–	–
Current leaf lower surface color	–	Light green (337)	Green (181)	Dark green (3)	–	–	–	–	–
Current branch leaf apex shape	–	Ronud (25)	Acute (496)	–	–	–	–	–	–
Fruit yield	–	Low (147)	Moderate (222)	High (152)	–	–	–	–	–
Ripening date	–	Late May (145)	Early June (45)	Mid-June (40)	Late June (65)	Early July (130)	Mid July (35)	Late July (61)	–
Exocarp splitting	Absent (37)	Present (484)	–	–	–	–	–	–	–
Fruit skin retention	Absent (256)	Present (265)	–	–	–	–	–	–	–
Fruit stigma retention	Absent (373)	Present (148)	–	–	–	–	–	–	–
Nut apex shape	–	Round (142)	Semi-round (137)	Acute (242)	–	–	–	–	–
Nut base shape	–	Round (338)	Semi-round (28)	Smooth (155)	–	–	–	–	–
Nut symmetry	Absent (91)	Present (430)	–	–	–	–	–	–	–
Nut shape	–	Round (21)	Hearty (13)	Oval (97)	Elongated oval (7)	Ovate (150)	Solvent (39)	Lacrimal (165)	Elongated lacrimal (29)
Position of maximum transverse diameter	–	Based (47)	Near base (341)	Center (133)	–	–	–	–	–
Shell surface pubescence	Absent (264)	Low (157)	Moderate (84)	High (16)	–	–	–	–	–
Shell hardness	–	Low (53)	Moderate (237)	High (231)	–	–	–	–	–
Shell color intensity	–	Cream (66)	Cream-brown (4)	Light brown (180)	Brown (179)	Dark brown (92)		–	–
Suture opening of the shell	Absent (511)	Present (10)	–	–	–	–	–	–	–
Shell ornamentation present	Absent (330)	Present (191)	–	–	–	–	–	–	–
Marking of outer shell	Absent (335)	Low (100)	Moderate (63)	High (23)	–	–	–	–	–
Shell ornamentation	Absent (335)	Scattered colored dots (186)			–	–	–	–	–
Shell abdominal line shape	–	Blade/low (349)	Blade/moderate (137)	Blade/high (35)	–	–	–	–	–
Shell back line shape	–	Studs/low (444)	Studs/moderate (8)	–	Smooth (54)	Embossed (15)	–	–	–
Shell abdominal line color	–	White (40)	Cream (119)	Cream-brown (7)	Light brown (118)	Brown (138)	Dark brown (80)	Black brown (19)	–
Shell back line color	–	White (40)	Cream (91)	Cream-brown (7)	Light brown (154)	Brown (158)	Dark brown (58)	Black brown (13)	–
Shell short furrows starting from base	Absent (276)	Present (245)	–	–	–	–	–	–	–
Kernel shape	–	Round (1)	Hearty (13)	Oval (57)	Elongated oval (7)	Ovate (331)	Solvent (10)	Lacrimal (97)	Elongated lacrimal (5)
Kernel color intensity	–	Cream (28)	Light brown (332)	Brown (160)	Dark brown (1)	–	–	–	–
Kernel shriveling	Absent (244)	Low (182)	Moderate (61)	High (34)	–	–	–	–	–
Kernel taste	–	–	Bitter (321)	Relatively bitter (170)	Relatively sweet (29)	Sweet (1)	–	–	–

Tree growth vigor was high in most accessions (259) (Table 3). Trunk diameter was moderate and then high in the majority of accessions (238 and 155, respectively). The stem diameter in *P. scoparia* is very important for the production of gum and resin. It has been reported that most resin-producing plants form resin-producing ducts for self-defense^20^. One of the most important reasons that wild almond trees show high resistance to pests and diseases can be attributed to the unique feature of gum production in them^21^. In addition, hydraulic conductivity in most plant species is enhanced by increasing the thickness of stems and branches. The movement of water and nutrients needed towards the fruit is done better by the thick branches, which increases the growth and quality of the fruit^22^.

Annual branch color was light green in 356, green in 299, dark green in 64, and brown in 3 accessions. Also, current branch color in summer was predominantly light green (427 accessions) and then green (245), while it was dark green in 31, crimson in 12, and purple-green in 7 accessions. The leaves of *P. scoparia* normally fall in early summer and then the green branches continue photosynthesis to provide carbohydrates for root and branch growth and development for the rest of the growing season. In this case, the green branches compensate for the lack or absence of leaves. Considering that cytokinins and gibberellins are produced in the root^23^, the transfer of these substances to the branches, instead of forming new leaves, causes internode elongation in *P. scoparia*^3,4^.

The range of related characters of leaves on the annual branch (branch of the previous year) was as follows: leaf length: 8.11–54.35 mm, leaf width: 1.23–10.15 mm, petiole length: 0.60–9.55 mm, and petiole width: 0.25–1.10 mm. The range of related characters of leaves on the current branch was as follows: leaf length: 8.00–40.25 mm, leaf width: 0.74–10.15 mm, petiole length: 0.46–10.31 mm, and petiole width: 0.30–0.91 mm (Table 2). In general, leaf area in *P. scoparia* is low, or in other words, its leaves are small, which indicates its greater adaptation to drought stress. Previous studies also reported that reduction of leaf area is an initial response of plant adaptation to drought conditions. Considering that the leaves of *P. scoparia* fall in early summer and the green shoots continue photosynthesis, this species can be a better choice as a rootstock. Also, the pubescence amount on the upper and lower surfaces of *P. scoparia* leaves is high, which is one of the responses to improve resistance to drought stress^24^.

Although the leaf size of *P. scoparia* is smaller, it is interesting that the dry matter content in this species is higher than that of domestic almonds. Dry and fresh weight in a plant determines its biomass production^25^. Interestingly, the cheapest and easiest method to track the performance and adaptation of plants to drought is to measure the accumulation of ash and mineral content^26^. It has been reported that plants with more dry matter have higher yields under salinity-stress conditions^27^. Previously, positive and significant correlations between leaf ash content and yield^28,29^ and also between leaf life span and dry matter content have been reported^30^.

The range of fruit stalk length and diameter was 1.85–5.53 mm and 0.88–3.48 mm, respectively. The range of nut-related characters was as follows: nut length: 9.72–22.87 mm, nut width: 5.81–15.54 mm, nut thickness: 5.67–12.00 mm, and nut weight: 0.18–0.99 mm. The suture opening of the shell was absent in 511 out of 521 accessions studied. The well-sealed shell is common in *P. scoparia* and is reported to be more resistant to fungus and insect infestation^31^. This offers new opportunities in breeding already not readily available in domesticated almond genetic resources. Shell thickness ranged from 0.40 to 1.76 mm. The presence of a relatively high variation in shell thickness among populations of *P. scoparia* offers the opportunity to select thin-shell nuts, which is important for the local production of this species as a nut crop^32^.

The range of kernel-related characters was as follows: kernel length: 6.83–19.23 mm, kernel width: 4.28–10.32 mm, kernel thickness: 2.16–7.52 mm, and kernel weight: 0.03–0.37 g. Empty nuts were observed in 79 out of 521 accessions. Variations in kernel size and the occurrence of empty nuts can be due to variations in humidity and rainfall occurring in the natural habitats. When the almond species are subjected to drought stress, they will start to use the kernel moisture which then results in the shrinking of the kernel and the decrease in nut size^3,33^. Kester et al.^34^ observed a highly significant effect of the environment on the occurrence of empty nuts, while Sanchez-Perez et al.^35^ indicated a diminutive annual variation in this particular trait.

**Figure 2:**
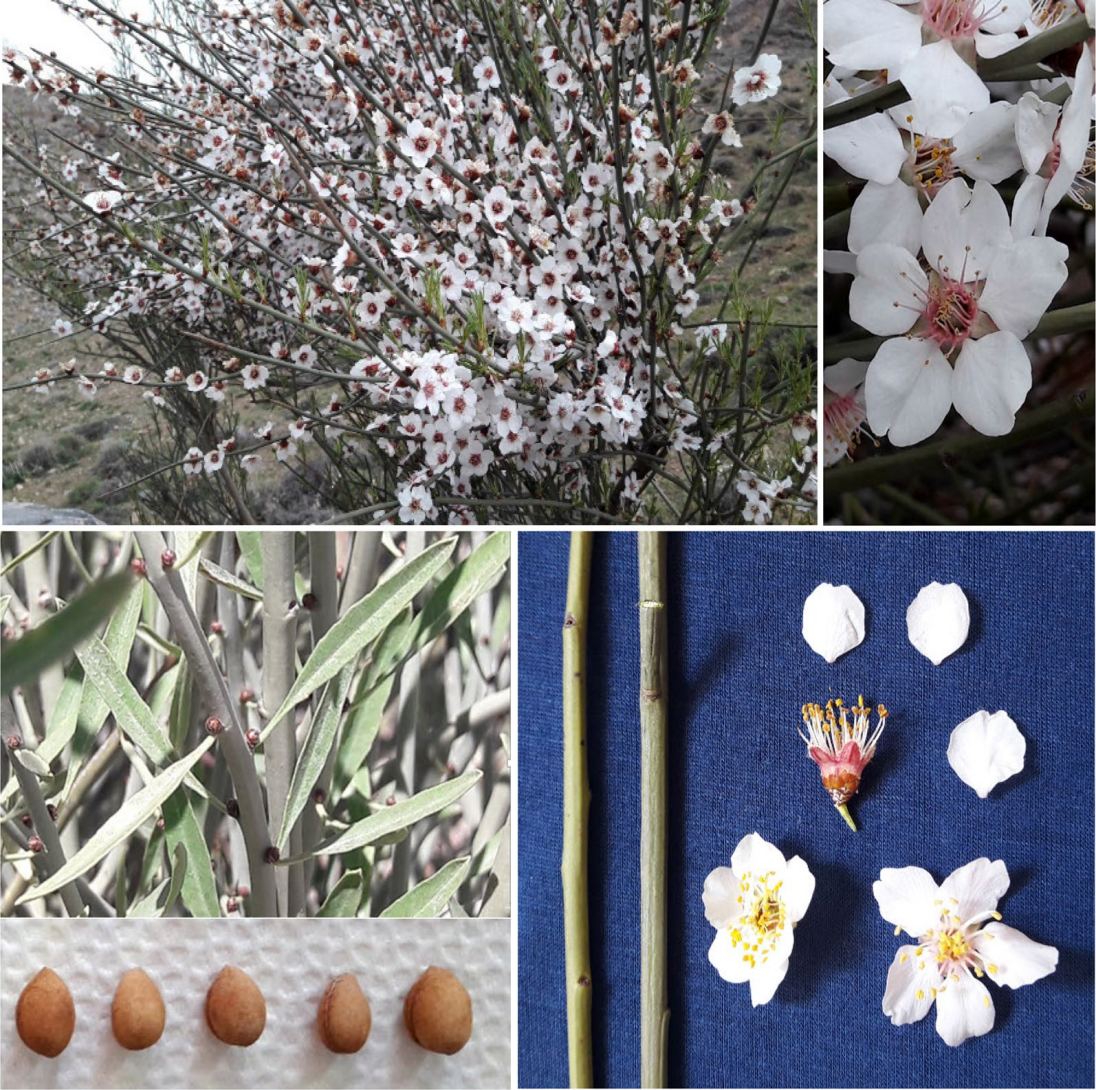
The leaves, flowers, and nuts of *P. scoparia* accessions studied.

Many the almond characteristics are genetically controlled^36^. Also, the differences in the characters of accessions of different areas could be mainly because of the wider geographic regions and climatic zones covered in this study. Another reason behind these differences could arise from the variation in climatic conditions, especially in rainfall. The variation in annual precipitation is very common in the arid and semi-arid climate of Iran^33^. The pictures of leaves, flowers, and nuts of *P. scoparia* accessions studied are shown in Fig. 2.

There were significant correlations between some characters (Table 4). Sepal length showed positive and significant correlations with peduncle length (*r* = 0.24), petal length (*r* = 0.60), petal width (*r* = 0.54), hypanthium length (*r* = 0.34), hypanthium diameter (*r* = 0.55), and sepal width (*r* = 0.73). Annual branch leaf length exhibited positive and significant correlations with annual branch leaf width (*r* = 0.43), annual branch petiole length (*r* = 0.57), and annual branch petiole width (*r* = 0.48). Current branch leaf length was positively and significantly correlated with current branch leaf width (*r* = 0.47), current branch petiole length (*r* = 0.71), and current branch petiole width (*r* = 0.51), in agreement with the previous findings^3,11,19,24,33,37^.

**Table 4:** Simple correlations between the quantitative morphological variables utilized in the studied *P. scoparia* accessions.

character	PedLe	PedWi	PetLe	PetWi	HyLe	HyDi	SepLe	SepWi	StLe	AnBrLLe	AnBrLWi	AnBrPetLe	AnBrPetWi	CuLLe	CuLWi	CuPetLe	CuPetWi	FrStaLe	FrStaDi	NuLe	NuWi	NuTh	NuWe	SheTh	KeLe	KeWi	KeTh	KeWe
PedLe	1																											
PedWi	0.24*	1																										
PetLe	0.17	0.14	1																									
PetWi	0.09	0.06	0.81**	1																								
HyLe	0.14	0.06	0.23*	0.22*	1																							
HyDi	0.07	0.05	0.58**	0.66**	0.21*	1																						
SepLe	0.24*	0.12	0.60**	0.54**	0.34**	0.55**	1																					
SepWi	0.20*	0.03	0.60**	0.68**	0.31**	0.66**	0.73**	1																				
StLe	0.19	− 0.16	0.21*	0.12	0.23*	0.08	0.26**	0.21*	1																			
AnBrLLe	0.13	− 0.13	− 0.04	0.08	− 0.04	0.12	0.04	0.11	0.08	1																		
AnBrLWi	− 0.09	0.05	− 0.09	− 0.03	− 0.12	− 0.05	− 0.08	− 0.07	− 0.14	0.43**	1																	
AnBrPetLe	0.06	0.00	− 0.15	− 0.11	0.05	− 0.08	− 0.10	− 0.08	0.07	0.57**	0.40**	1																
AnBrPetWi	0.02	− 0.03	− 0.10	− 0.12	− 0.09	0.03	− 0.09	− 0.03	0.07	0.48**	0.27**	0.36**	1															
CuLLe	0.06	− 0.03	− 0.16	− 0.10	− 0.01	− 0.05	− 0.11	− 0.09	0.02	0.60**	0.22*	0.47**	0.32**	1														
CuLWi	− 0.03	0.20*	− 0.13	− 0.10	− 0.10	− 0.16	− 0.16	− 0.22*	− 0.17	0.24*	0.55**	0.37**	0.14	0.47**	1													
CuPetLe	0.08	− 0.01	− 0.23*	− 0.15	0.08	− 0.11	− 0.15	− 0.15	0.04	0.43**	0.26**	0.55**	0.21*	0.71**	0.50**	1												
CuPetWi	0.00	− 0.07	− 0.21*	− 0.25**	− 0.09	− 0.11	− 0.13	− 0.12	0.06	0.37**	0.24*	0.32**	0.55**	0.53**	0.24*	0.45**	1											
FrStaLe	0.12	0.15	0.06	0.05	− 0.06	0.04	− 0.04	0.03	0.01	0.11	0.00	0.08	0.06	0.12	0.03	0.13	0.03	1										
FrStaDi	− 0.12	0.29**	− 0.01	− 0.01	− 0.02	− 0.02	− 0.08	− 0.12	− 0.25**	− 0.24*	0.14	− 0.08	− 0.15	− 0.15	0.22*	− 0.05	− 0.10	0.01	1									
NuLe	0.03	0.24*	− 0.21*	− 0.25**	− 0.15	− 0.25**	− 0.09	− 0.25**	− 0.20**	0.00	0.08	0.03	− 0.04	0.02	0.23*	0.01	− 0.07	0.04	0.22*	1								
NuWi	− 0.07	0.10*	− 0.09	− 0.12	− 0.08	− 0.11	0.02	− 0.07	− 0.13	− 0.02	0.06	− 0.01	0.11	− 0.03	0.05	− 0.07	0.03	0.01	0.15	0.65**	1							
NuTh	− 0.11	0.05	− 0.16	− 0.17	− 0.07	− 0.09	− 0.05	− 0.10	− 0.20**	− 0.01	0.11	0.00	0.07	0.07	0.13	0.04	0.10	0.00	0.20*	0.50**	0.75**	1						
NuWe	− 0.04	0.14	− 0.17	− 0.21*	− 0.11	− 0.20*	− 0.08	− 0.18	− 0.15	0.04	0.06	0.05	0.11	0.05	0.11	0.01	0.05	0.03	0.18	0.77**	0.84**	0.70**	1					
SheTh	0.06	− 0.01	0.01	− 0.08	− 0.04	− 0.03	0.10	0.05	− 0.02	− 0.06	− 0.10	− 0.08	0.29**	− 0.10	− 0.20*	− 0.20*	0.16	− 0.04	− 0.07	0.22*	0.52**	0.33**	0.44**	1				
KeLe	0.03	0.14	− 0.20**	− 0.24*	− 0.20*	− 0.29**	− 0.14	− 0.28**	− 0.08	0.00	0.03	0.00	0.00	0.00	0.12	− 0.03	− 0.05	0.07	0.08	0.82**	0.55**	0.35**	0.72**	0.19	1			
KeWi	− 0.04	0.01	− 0.17	− 0.24*	− 0.20*	− 0.27**	− 0.13	− 0.23*	− 0.06	− 0.09	0.02	0.00	0.02	− 0.08	− 0.01	− 0.08	− 0.04	0.02	0.09	0.55**	0.71**	0.52**	0.73**	0.37**	0.68**	1		
KeTh	− 0.10	− 0.01	− 0.24*	− 0.26**	− 0.22*	− 0.22*	− 0.33**	− 0.34**	− 0.02	− 0.10	0.03	− 0.01	− 0.03	0.07	0.06	0.10	0.08	0.08	0.01	0.05	0.04	0.25**	0.16	− 0.21*	0.27**	0.37**	1	
KeWe	− 0.07	0.05	− 0.30**	− 0.35**	− 0.26**	− 0.34**	− 0.28**	− 0.37**	− 0.08	− 0.01	0.05	0.06	0.09	0.07	0.10	0.06	0.09	0.09	0.05	0.57**	0.54**	0.42**	0.69**	0.16	0.75**	0.78**	0.58**	1

For an explanation of the morphological character symbols, see Table 2.

*,**Correlation is significant at *P* ≤ 0.05 and 0.01 levels, respectively.

Nut weight was positively and significantly correlated with nut length (*r* = 0.77), nut width (*r* = 0.84), nut thickness (*r* = 0.70), and shell thickness (*r* = 0.44). Kernel weight exhibited positive and significant correlations with nut length (*r* = 0.57), nut width (*r* = 0.54), nut thickness (*r* = 0.42), nut weight (*r* = 0.69), kernel length (*r* = 0.75), kernel width (*r* = 0.78), and kernel thickness (*r* = 0.58), in agreement with the previous findings^3,11,19,24,37,38^.

PCA placed the traits in 26 components that explained 72.44% of the total variance. PC1 accounted for 6.10% of the total variance and showed significant correlations with petal length, petal shape, petal width, hypanthium diameter, sepal length, and sepal width. Nut length, nut thickness, nut width, nut weight, kernel weight, kernel width, and kernel length were placed in PC2 and explained 6.06% of the total variance. Tree form, trunk type, tree height, and trunk diameter were placed in PC3 and explained 4.58% of the total variance (Table 5). It has been reported that fruit-related traits are important for distinguishing accessions of almond species^9,33^.

**Table 5:** Eigenvectors for the main variables for the first three principal component axes from PCA of the morphological characters in the studied *P. scoparia* accessions.

Character	Component		
	1	2	3
Petal shape	**− 0.66**	0.08	− 0.06
Petal length	**0.77**	− 0.12	− 0.05
Petal width	**0.85**	− 0.16	− 0.05
Hypanthium diameter	**0.75**	− 0.15	− 0.12
Sepal length	**0.74**	− 0.02	− 0.15
Sepal width	**0.81**	− 0.12	− 0.20
Tree form	− 0.23	0.06	**0.80**
Tree height	− 0.14	0.04	**0.81**
Trunk type	0.11	0.01	**− 0.81**
Trunk diameter	− 0.02	0.12	**0.73**
Nut length	− 0.13	**0.76**	0.14
Nut width	0.01	**0.88**	0.02
Nut thickness	− 0.12	**0.73**	0.02
Nut weight	− 0.07	**0.92**	0.05
Kernel length	− 0.12	**0.78**	0.10
Kernel width	− 0.13	**0.86**	− 0.03
Kernel weight	− 0.25	**0.79**	0.05
Total	6.10	6.06	4.58
% of variance	6.10	6.06	4.58
Cumulative %	6.10	12.16	16.74

Bold values indicate the characteristics that most influence each PC.

**Figure 3:**
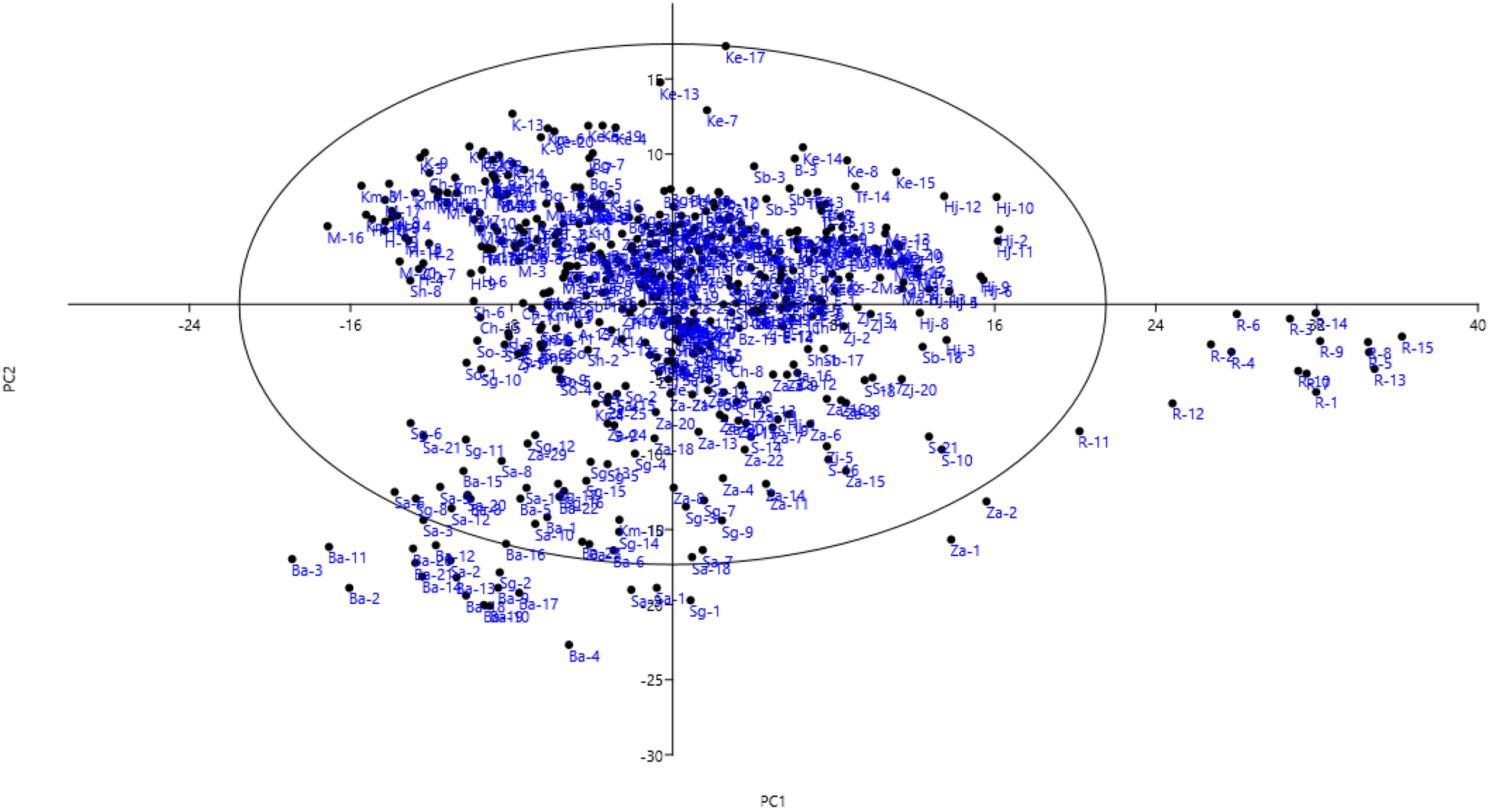
Scatter plot for the studied *P. scoparia* accessions based on PC1/PC2. The symbols represent the accessions of each area in the plot (for an explanation of accession symbols, see Table 1).

In the scatter plot, the accessions were widely distributed on the plot level (Fig. 3). The results of the plot showed that the accessions have considerable variation so that a large number were placed outside the oval, which indicates their high differences with other accessions in terms of traits in PC1 and PC2.

**Figure 4:**
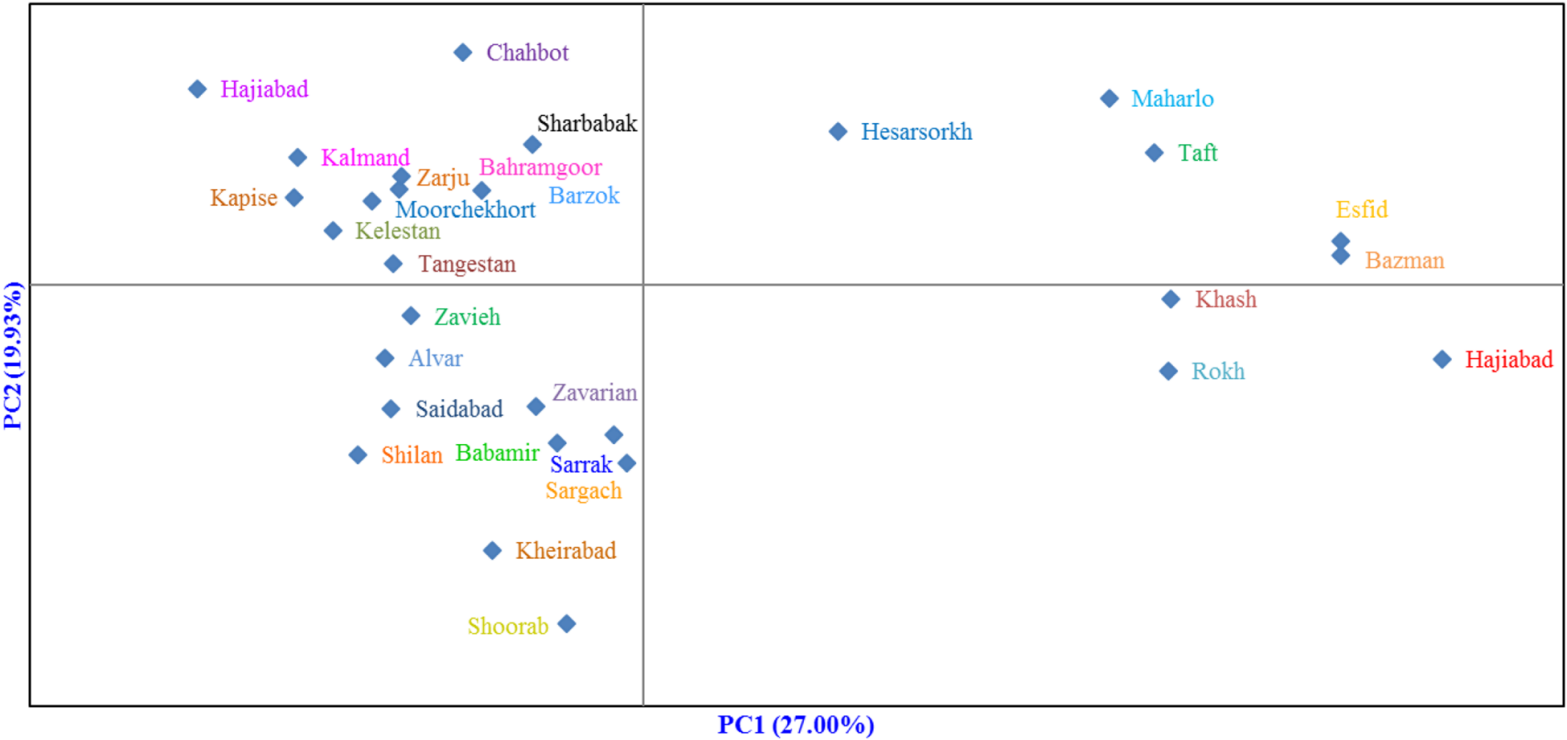
Bi-plot for the studied populations of *P. scoparia* based on the morphological characters.

The dendrogram created through Ward's method and Euclidean distance divided the accessions into two groups, each group having several subgroups, which indicates the high variation among accessions (not shown). Also, the studied 29 populations were placed into four groups in the bi-plot generated with PCA of population analysis (Fig. 4). Maharlo, Taft, Hesarsorkh, Bazman, and Esfid populations were placed into the first group, and Khash, Hajiabad, and Rokh populations were placed in the second group. Also, 11 populations, including Chahbot, Sharbabak, Hajiabad, Kalmand, Bahramgoor, Zarju, Barzok, Moorchekhort, Kapise, Kelestan, and Tangestan formed the third group, while the fourth group consisted of the rest 10 populations, including Zavieh, Alvar, Saidabad, Zavarian, Sarrak, Babamir, Shilan, Sargach, Kheirabad, and Shoorab.

The studied accessions showed considerable variation in terms of the measured traits within and among populations. This variation is due to cross-pollination, natural hybridization, cross-incompatibility, propagation by seeds, gene flow, and exchange of plant material between the study areas^39^. The traditional method of propagation and distribution of almonds is through seeds, which has caused the differentiation of traits and increased diversity over time. Also, the exchange of almond germplasm has been done in this country for millennia due to communication and interactions, which has caused interbreeding between populations. Thus, it is possible to justify the grouping of accessions of some distant populations^40–42^. Also, the dissimilarity between accessions of the species denotes the capability of generating new progenies and producing different associations or segregations of genes, thereby facilitating a partial removal of former linkages or the creation of new ones that can be applied in both classical and modern breeding methods. To generate new progenies in a subsequent generation (with new linkage groups or new population properties), it is a common practice to use distant genotypes^40–42^.

Frost resistance is a major breeding goal for almond cultivars in many production areas because of their early flowering time during late winter and early spring. Some accessions of *P. scoparia* showed late flowering time. The possibility of use of almond related species with a very late-flowering date (high chilling requirements) to develop new cultivars with late-flowering would not only reduce frost damage, but reduce disease damage if flowering is delayed beyond the rainy season, and would allow more efficient use of increasingly scarce insect pollinators^43^.

## Conclusion

A wide range of variations was detected within and among the populations studied of *P. scoparia*. The obtained results can be important for the management and protection of the gene pool. Also, these findings can be used to develop and introduce new rootstocks for almonds and other stone fruits. The traits, such as late flowering time, suitable trunk diameter, low tree height, suitable tree growth vigor, small leaf size, high nut weight, and high kernel weight are desirable traits that can be considered in almond breeding programs. Also, local cultivation of *P. scoparia* can be considered for the production of nuts and oil extraction. By using crosses between accessions of different regions, it is possible to increase the amount of variability in different traits of wild almonds.

## Data Availability

The findings supporting the present study, when reasonable request, are available from the corresponding author.
